# Supplementary material for: Antiviral Effect of Antimicrobial Peptoid TM9 and Murine Model of Respiratory Coronavirus Infection
Source: Pharmaceutics. 2024 Mar 27;16(4):464. doi: 10.3390/pharmaceutics16040464 (PMC11054490; doi:10.3390/pharmaceutics16040464)
Supplement: Supplementary file 1 [file pharmaceutics-16-00464-s001.zip › Figure S2.pdf]

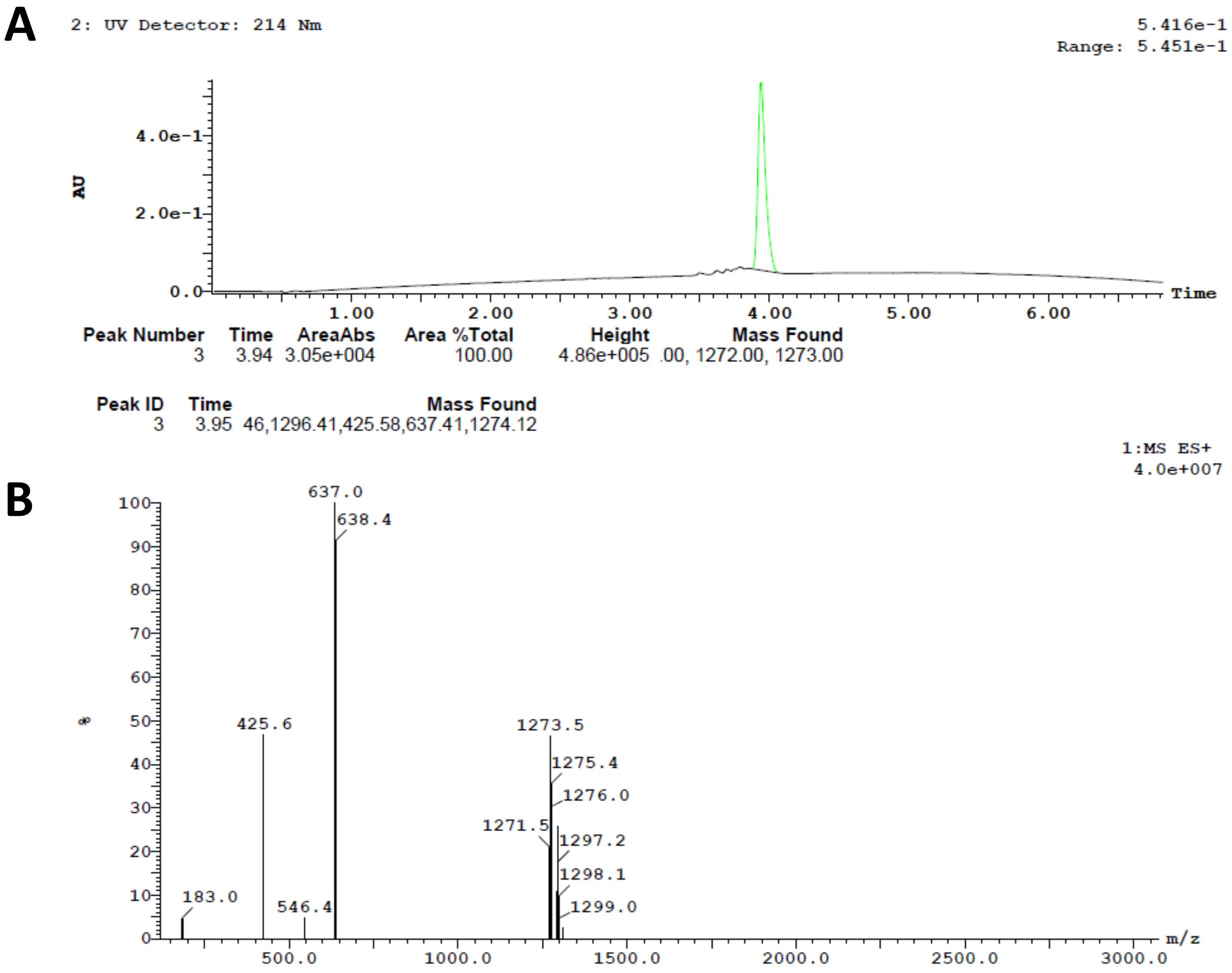

Figure S2. LC/MS trace of TM9. LC/MS trace of TM9 using a 5-95% gradient of acetonitrile + 0.1% TFA over 6.8 minutes. The retention time of TM9 was 3.94 minutes: MS (ESI$^+$, [M]$^+$, $C_{64}H_{92}Br_2N_{10}O_7$): 1271.50.
