# Supplementary figures and images for: Antiviral Effect of Antimicrobial Peptoid TM9 and Murine Model of Respiratory Coronavirus Infection
Source: Pharmaceutics. 2024 Mar 27;16(4):464. doi: 10.3390/pharmaceutics16040464 (PMC11054490; doi:10.3390/pharmaceutics16040464)

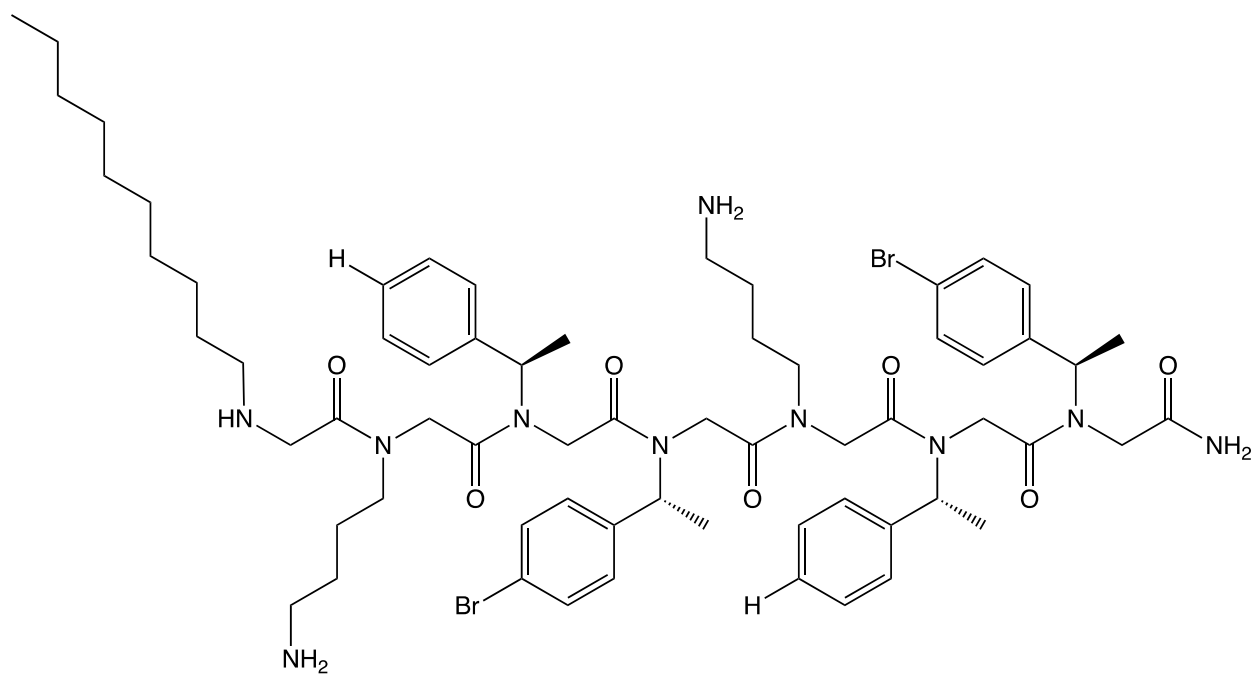

**Figure S1. Structure of TM9.** Structure of TM9

Supplement: Supplementary file 1 [file pharmaceutics-16-00464-s001.zip › Figure S1.pdf]
